# Polymorphisms of the porcine cathepsins, growth hormone-releasing hormone and leptin receptor genes and their association with meat quality traits in Ukrainian Large White breed

**DOI:** 10.1007/s11033-016-3977-z

**Published:** 2016-04-13

**Authors:** Viktor Balatsky, Irina Bankovska, Ramona N. Pena, Artem Saienko, Tetyana Buslyk, Sergii Korinnyi, Olena Doran

**Affiliations:** Laboratory of Genetics, Institute of Pig Breeding and Agro-Industrial Production, National Academy of Agricultural Sciences of Ukraine, Shvedska Mogila 1, Poltava, 36013 Ukraine; Department of Animal Production, University of Lleida-Agrotecnio Centre, Av Alcalde Rovira Roure 191, 25198 Lleida, Spain; Department of Biological, Biomedical and Analytical Sciences, Faculty of Health and Applied Sciences, Centre for Research in Biosciences, University of the West of England, Frenchay Campus, Coldharbour Lane, Bristol, BS16 1QY UK

**Keywords:** Pig, Polymorphisms, Genotyping, Association study, Meat quality traits

## Abstract

Cathepsins, growth hormone-releasing hormone (*GHRH*) and leptin receptor (*LEPR*) genes have been receiving increasing attention as potential markers for meat quality and pig performance traits. This study investigated the allele variants in four cathepsin genes (*CTSB*, *CTSK, CTSL, CTSS*), *GHRH* and *LEPR* in pure-bred Ukrainian Large White pigs and evaluated effects of the allele variants on meat quality characteristics. The study was conducted on 72 pigs. Genotyping was performed using PCR–RFLP technique. Meat quality characteristics analysed were intramuscular fat content, tenderness, total water content, ultimate pH, crude protein and ashes. A medium level of heterozygosity values was established for *GHRH* and *LEPR* genes which corresponded to very high levels of informativeness indexes. Cathepsins *CTSL*, *CTSB* and *CTSK* had a low level of heterozygosity, and *CTSS* did not segregate in this breed. Association studies established that intramuscular fat content and tenderness were affected by the allele variance in *GHRH* and *LEPR* but not by *CTSB* and *CTSL* genes. The *GHRH* results could be particularly relevant for the production of lean prime cuts as the A allele is associated with both, a lower meat fat content and better tenderness values, which are two attributes highly regarded by consumers. Results of this study suggest that selective breeding towards *GHRH*/AA genotype would be particularly useful for improving meat quality characteristics in the production systems involving lean Large White lines, which typically have less than 2 % intramuscular fat content.

## Introduction

During the last decades, the international pig industry has been focusing on the selective breeding towards genotypes with high growth rate, improved feed efficiency and increased meat content. Unfortunately, this was accompanied by reduction of intramuscular fat and water holding capacity, which had deteriorating effect on meat eating quality traits such as flavour and juiciness [1]. The industry has been employing a number of strategies to enhance intramuscular fat content and improve meat eating quality characteristics. This includes manipulation of pig diets and revised management procedures during animals’ transportation and slaughter [2, 3]. In parallel, increasing attention has been paid to identifying genetic markers for desirable combination of meat quality and animal performance traits. A number of Quantitative Trait Loci (QTLs) associated with pig meat quality traits and/or carcass composition have been reported (reviewed in [4]) and several cathepsin genes have been identified as potential genetic markers among positional candidate genes. Cathepsins are a family of peptidase enzymes which are present mainly in the lysosomes of the skeletal muscles using actin, myosin and associated proteins as substrates for their reaction [5]. Therefore, cathepsins could play an important role in the post-*mortem* meat tenderisation [6]. It has been demonstrated that polymorphisms in porcine cathepsin genes are associated with daily gain, backfat thickness, lean content and intramuscular fat content [7–13].

In parallel with cathepsins, the Growth Hormone Releasing Hormone (*GHRH*), which controls muscle growth and development, has been considered as a candidate gene for pig performance and meat quality traits. Polymorphisms in *GHRH* gene have been shown to be associated with backfat thickness, daily gain, carcass meat content, meat colour and water-holding capacity [14, 15]. Similarly, mutations in the leptin receptor (*LEPR*) have been associated with traits directly related to fat deposition, such as backfat thickness, intramuscular fat content, lean content and average daily gain [16–18].

The mechanisms regulating meat quality traits and pig performance are breed-specific due to interaction with genetic background that composes each population [19]. Therefore, it is important to evaluate the strengths of associations between candidate genetic markers and meat quality traits in the breeds and pig lines intended for specific markets. In this regard, Large White pigs are widely used for crossbreeding and to generate new production lines in 117 countries across the world [20, 21]. During the last decades, an increasing attention has been paid to traditional Ukrainian pig breeds and pig lines including Ukrainian Large White. This breed was created in the end of 20th century on the basis of English Large White pig breed using a complex selection process aiming to improve meat quality traits. Ukrainian Large White pigs have multiple pregnancies with an average litter size of 11.6 piglets and a daily weight gain 800–850 g/day. They also have strong body built and are well adapted to difficult climate conditions. Ukrainian Large White pigs have been widely used for production of “organic” pork and outdoor rearing. Depending on the live weight at the slaughter time, Ukrainian Large White pigs can be used for production of a range of products, from lean meat and bacon to traditional Ukrainian “salo”, which is cured slabs of fatback.

In spite of a large number of publications on *GHRH* and *LEPR* polymorphisms, there is very limited and fragmental information on associations between there polymorphisms and meat quality and pig performance traits. Previous studies were carried out on common Large White populations from a number of European countries but not on Ukraine Large White pigs [8, 9, 15, 17]. Furthermore, so far no studies have tested associations between cathepsin genes, *GHRH* and *LEPR* and meat quality traits such a protein content, tenderness, ashes, and total water in Large White pigs.

The aim of the present study was to investigate the strength of associations between polymorphisms in the cathepsin genes (*CTSB, CTSK, CTSL,* and *CTSS*), *GHRH* and *LEPR* with meat quality traits in purebred Ukrainian Large White pigs. Specific objectives were: (i) to identify the allele variants of *CTSB*, *CTSK, CTSL, CTSS, GHRH* and *LEPR* in the Ukrainian Large White pigs, and (ii) to evaluate effects of these allele variants on meat quality traits.

## Materials and methods

### Animals

The study was conducted on 72 pigs of Ukrainian Large White breed. The authors recognise that this number is lower when compared to other association studies. This was due the fact that the size of Ukrainian Large White population is relatively small (around 500 pure bred pigs in Ukraine) and it is not easy to access these animals. Experimental protocol was approved by the Scientific Committee of the Institute of Pig Breeding and Agro-Industrial Production, National Academy of Agricultural Sciences, Ukraine. All the procedure related to animal handling and slaughter were conducted in accordance with the European Convention for the Protection of Vertebrate Animals used for Experimental and Others Scientific Purposes [22].

The study used castrated males or females in approximately 1:1 ratio. The animals were raised at the facilities of the Ukrainian Academy of Agricultural Sciences, Ukraine, fed a standard commercial finishing diet ad libitum from the live weight of 40 kg, were handled by the same staff, and were slaughtered at the average live weight of 109 kg in one season, at the same time of the day and under the same conditions. In the association studies, the gender of animals was present as a random factor. The association studies design was based on the approach described by [23].

During the growth phase between 40 and 60 kg (live weight), the feed contained per dry matter: 12.9 MJ/kg of net energy, 19.1 % of crude protein and 1.14 % of lysine. During the growth phase between 60 and 90 kg (live weight), the feed contained 12.8 MJ/kg of net energy, 18.0 % of crude protein and 1.0 % of lysine. During the finishing phase (live weight 90–109 kg), the feed contained 12.6 MJ/kg of net energy, 17.1 % of crude protein and 0.8 % of lysine. The feed was produced by Poltava Feed Mill (Poltava, Ukraine).

Samples of blood (1 ml) were collected when the pigs reached the weight of 109 kg. The blood was collected in the morning before feeding. The blood samples were mixed with 0.05 M EDTA and stored up to 7 days at +4 °C until used for DNA isolation.

### Analysis of meat quality traits

The pigs used in the association studies were tested for the c.1843 C>T mutation in the ryanodine receptor gene associated with pig meat defects [24]. All the animals had genotype CC, e.g. the mutant allele variant was absent.

Ultimate pH was measured by a portable digital LF-Meter “LF-Star CPU-Pistole” (Ing.-Büro & Klassifizierungsservice Rudolf Matthäus, Klausa, Germany) after cooling the carcasses for 48 h at +2 to +4 °C. The analyses were conducted on samples from *M. longissimus dorsi* collected between the 10th and 12th thoracic vertebra. The samples were collected from the same position on the right side of the carcasses.

The total water content was determined by drying of meat samples at 105 °C to constant weight; ash analysis was carried out by burning the meat samples in a muffle furnace at 550° C; crude protein content was analysed by Kjeldahl’s method, and intramuscular fat content was determent by Soxhlet’s method as described in Official Methods of Analysis [25].

Meat tenderness was determined using the protocol for Warner–Bratzler shear force analysis [26]. For each sample, two cylindrical cores (8 cm long and 2.5 cm in the diameter) were taken in parallel to the longitudinal orientation of the muscle fibres. The cylindrical cores were shredded by a cutting blade at a velocity 200 mm/min at six points. The time of shredding was recorded for each point and averages were calculated to determine the shear force value per sample.

### PCR amplification

Genomic DNA was isolated from blood by the sorbent method using Diatom™ DNA Prep 100 kit (Isogen, Russia, Moscow) following the manufacturer’ instructions with guanidine thiocyanate as the lysis reagent. Polymerase Chain Reaction (PCR) for Restriction Fragment Length Polymorphism Analysis of PCR-Amplified Fragments (PCR–RFLP) genotyping was carried out in a final volume of 25 µl which contained 200 nm of corresponding forward and reverse primers, 1.5, 2.0 or 2.5 mM MgCl_2_, 0.25 mM of each of the dNTP and 1 unit of the recombinant Taq DNA Polymerase (Fermentas, Vilnius, Lithuania).

### Genotyping

*CTSB*, *CTSL*, *CTSS* and *GHRH* were genotyped using PCR–RFLP technique as described by [9, 27, 28]. *CTSL* was genotyped on the g.143T>C SNP (NCBI accession number rs 81212773); *CTSS* was genotyped on the g.171G>A SNP (NCBI accession number rs 331232519); *CTSB* was genotyped on the g.72A>C SNP (EMBL accession number AJ315558), and *GHRH* was genotyped on the *Alu*I polymorphic restriction site in exon 3.

For the *CTSL* restriction analysis, 5 µl of PCR product was digested overnight at 65 °C with 3 units of *Taq*I restriction enzyme (Fermentas) in the final volume of 25 µl of the reaction buffer. Digestion of the *CTSS* PCR product was conducted under conditions similar to that for *CTSL* but with the *Bse*NI endonuclease (Fermentas). The restriction analysis of *CTSB* amplification product was carried out by digestion with *Msp*I endonuclease (Fermentas), and the restriction analysis of *GHRH* amplification product was carried out using the restriction enzyme *Alu*I (Fermentas). The DNA fragments obtained by restrictive digestion, were separated on 4 % agarose gels, and DNA was visualised using ethidium bromide (0.5 µg/µl *CTSK* g.15G>A SNP (NCBI accession number rs 337183461) and *LEPR* c.1987C>T SNP (db SNP accession number ss 262803826) were genotyped using allelic discrimination and High Resolution Melting (HRM) methods respectively. For HRM, Luminaris Color HRM qPCR Master Mix (LifeTechnologies, Grand Island, NY, USA) was used. The HRM primers were designed using Primer3Plus program (http://www.bioinformatics.nl/cgi-bin/primer3plus/primer3plus.cgi) with the qPCR default settings, while the allelic discrimination primers and probes were designed as part of Life Technologies custom assays. Both analyses were conducted in CFX96 Touch™ Real Time PCR Detection System. The primers, probes and PCR conditions are given in Table 1.

**Table 1:** PCR primers, condition, and PRC-RELP patterns of different alleles of *CTSB,* *CTSL*, *CTSS*, *CTSK*, *GHRH* and *LEPR* genes in Ukrainian Large White pigs

Gene	Primer sequences (5′ → 3′)	PCR conditions			Genotyping method
		Length (bp)	Tm (°C)	MgCl_2_ (mM)	
*CTSB*	F: GTGGCCGGGTGGGTTTTA R: TCCTCCTGGTGCTGCTAATTCTGAC	139	55	2.0	PCR–RFLP (*Msp*I)
*CTSL*	F: TCACTGCCGTGAAGAATCAG R: GCAGAGCTGTAATGGCAAGA	380	64	2.5	PCR–RFLP (*Taq*I)
*CTSS*	F: AGAGAGCCAGAGGTTGCTCA R: GCAGGCAGAGCAAGCTAAA	280	58	1.5	PCR–RFLP (*Bse*NI)
*CTSK*	F: TTGGGCGATATGGTGAGTTGAG R: CATAAGAAAGGAACCAAGGCAAACA Probe-G: VIC-CAGCTCCT**G**GTCTATC-NFQ Probe-A: FAM-TCAGCTCCT**A**GTCTATC-NFQ	66	60	3.0	Allelic discrimination
*GHRH*	F: GTAAGGATGC(C/T)(A/G)CTCTGGGT R: TGCCTGCTCATGATGTCCTGGA	455	63	1.5	PCR–RFLP(*Alu*I):
*LEPR*	F: CAGAGGACCTGAATTTTGGAG R: CATAAAAATCAGAAATACCTTCCAG	94	60	3.0	HRM

### Statistical analysis

Allele frequencies, genotype frequencies, polymorphic information content (PIC), and levels of heterozygosity (observed heterozygosity, H_o_ and expected heterozygosity, H_e_) were calculated with GenAlEx 6.0. software [29]. Analysis of associations between genotypes and meat quality characteristics were conducted by one way ANOVA. Means between groups were compared with a two-tailed *t* test using JMP12 (SAS Inst. Inc., Cary, NC) and differences were considered significant at P < 0.05.

Additive (*A*) and Dominance (*D*) components were calculated using the following equations:

$$A = \overline{X}_{22} - \overline{X}_{11} ,\quad D = \overline{X}_{12} - \frac{{\overline{X}_{11} + \overline{X}_{22} }}{2}$$

where $\overline{X}_{11} ,\overline{X}_{12} ,\overline{X}_{22}$ are arithmetic mean value of productivity traits for genotypes 1/1, 1/2 and 2/2.

The effect of alleles 1 and 2 were estimated using the equations $$\begin{aligned} \alpha_{1} = m_{1} - \bar{X} \hfill \\ \alpha_{2} = m_{2} - \bar{X} \hfill \\ \end{aligned}$$ where

$$\begin{aligned} m_{1} &= p \cdot \overline{X}_{11} + q \cdot \overline{X}_{12} \hfill \\ m_{2} &= p \cdot X12 + q \cdot \overline{X}_{22} , \hfill \\ \end{aligned}$$

*p* and *q* are allelic frequency 1 and 2 respectively; and $\overline{X}$ is the total arithmetic mean value for each trait. Allelic substitution effects, $\frac{\alpha }{2}(1 \to 2)$ were calculated using the equation

$$\frac{\alpha }{2}(1 \to 2) = \frac{{\alpha_{2} - \alpha_{1} }}{2}$$

## Results and discussion

### Allele frequencies and heterozygosity of the cathepsin, *GHRH* and *LEPR* genes

The SNP polymorphisms of the cathepsin *CTSB*, *CTSL* and *CTSK* genes were segregating in the Ukrainian Large White population analysed. Overall, these three SNPs showed extreme allelic frequencies, with major allele frequency (MAF) ranging from 0.02 to 0.09 (Table 2). Major alleles were A for the *CTSB* g.72A>C SNP, C for the *CTSL* g.143C>T SNP and G for the *CTSK* g.15G>A SNP. Consequently, observed heterozygosity (H_o_) was low, indicating low genetic diversity at these loci. Expected (H_e_) heterozygosity values were in agreement with observed heterozygosity (H_o_), which indicates that these loci are in Hardy–Weinberg equilibrium. The narrow level of genetic diversity of these three loci is also shown by the low polymorphic information content (PIC) of these markers (Table 2). The allele frequencies determined in this study for the Ukrainian Large White pigs were similar to those observed by [7] for *CTSB* g.72A>C in Italian Large White pigs (MAF = 0.06 for allele C). According to [7], g.72A was also predominant in Landrace, Belgium Landrace, Duroc, Piétrain and Hampshire breeds. Also for *CTSL,* the results were consistent with previous data indicating the predominance of g.143C allele in Italian Large White, Italian Landrace, Piétrain, Belgian Landrace, Hampshire and Meishan [9]. Similarly, for *CTSK*, allele g.15G has also been detected as the predominant allele in Italian Large White pig [30].

**Table 2:** Genotypes, allele frequencies and heterozygosity for *CTSB, CTSL, CTSS, CTSK, GHRH* and *LEPR* genes in Ukrainian Large White pig breed

Gene	Genotype	N	Genotype frequency	Allele frequency		H_o_^a^	H_e_^b^	PIC^c^
				g.72A	g.72C			
*CTSB*	g.72AA	66	0.92					
	g.72AC	6	0.08	0.96	0.04	0.08	0.08	0.074
	g.72CC	–	0.00					

				g.143C	g.143T			
*CTSL*	g.143CC	59	0.82					
	g.143CT	13	0.18	0.91	0.09	0.18	0.16	0.150
	g.143TT	–	0.00					

				g.171G	g.171A			
*CTSS*	g.171GG	72	1.00					
	g.171GA	–	0.00	1.00	0.00	0.00	0.00	0.00
	g.171AA	–	0.00					

				g.15G	g.15A			
*CTSK*	g.15GG	70	0.97					
	g.15GA	2	0.03	0.98	0.02	0.03	0.04	0.038
	g.15AA	–	0.00					

				A	B			
*GHRH*	AA	5	0.07					
	AB	29	0.40	0.27	0.73	0.40	0.39	0.317
	BB	38	0.53					

				c.1987C	c.1987T			
*LEPR*	c.1987CC	32	0.44					
	c.1987CT	32	0.44	0.67	0.33	0.44	0.44	0.344
	c.1987TT	8	0.12					

^a^ Observed heterozygosity; ^b^ Expected heterozygosity; ^c^ Polymorphic information content

*N* number of animals

In contrast to the above, the *CTSS* G>A polymorphism at the g.171 site was not segregating in Ukrainian Large White pigs as all the animals had genotype GG, although it has been reported to segregate in Italian Duroc, Italian Landrace Hampshire, Belgium Landrace and Piétrain in the range of 0.63–0.95 for the allele g.171G [9, 11]. The absence of segregation might be due to a different origin of Ukrainian Large White which derived from English Large White pigs.

On the other hand, *GHRH* and *LEPR* polymorphisms segregated in Ukrainian Large White pigs at close to intermediate frequencies (Table 2). Allele B was the most prevalent for *GHRHAlu*I polymorphism (allelic frequency of 0.73), and allele C was the most prevalent for the *LEPR* c.1987C>TSNP (allelic frequency of 0.67). A previous study showed that the B allele in *GHRH* gene was the most prevalent in Large White and Meishan pigs used as first generation of the PiGMap reference families [28], which is in agreement with our results. As seen for the other SNPs, the observed and expected heterozygosity values were very similar. In our case, the PIC of both markers was very high (0.317 and 0.344, respectively), considering that the maximum PIC for a two-allele polymorphism is 0.375. This level of in formativeness is most favourable for undertaking association studies [31].

### Analysis of associations between *CTSB, CTSL*, *GHRH* and *LEPR* genes and meat quality characteristics

We have investigated the contribution of mutations in six candidate genes to several muscle attributes related to the quality of meat in Ukrainian Large White pigs. Large White breed is widely used worldwide for efficient meat production, and it is particularly appreciated for the high lean content in prime cuts. Four cathepsin genes (*CTSB*, *CTSL*, *CTSS*, and *CTSK*) have been investigated in this study. Cathepsins are proteases, which are involved in structural and biochemical changes that take place during *post*-*mortem* storage of meat [32]. Large differences in the activities of these enzymes have been detected among pig genotypes [33] with Large White pigs showing a particularly high activity of cathepsin B in *biceps femoris*. Association analysis between the genotype of the cathepsin genes and meat quality-related traits was performed for *CTSB* and *CTSL* (but not for *CTSK*) loci, as only two pigs were heterozygous for the *CTSK* polymorphism.

 We did not established any significant relationship between the g.72A>C *CTSB* polymorphism and total protein content, water, ashes, intramuscular fat content and muscle shear force (Table 3). The protein content in the muscles of Ukrainian Large White pigs was in a range of 20.5–21.8 g/100 g of tissue which is slightly higher when compared to the muscle protein level in Large White pigs (19.5–20.0 mg/100 g of tissue) reported by [34]. At the same time, the Ukrainian Large White population had a lower average intramuscular fat content of 1.61 g/100 g of tissue when compared to a commercial Large White cross breed (1.91 g/100 g of tissue [35]). There was a tendency towards a lower muscle pH value at 48 h *post*-*mortem* in g.72AA pigs when compared to g.72AC, but these differences were not statistically significant (*P* = 0.099). The average pH value in our study was 5.53 which is comparable to pH values in purebred Large White and Duroc × Landrace × Large White cross breed (5.88 and 5.57 respectively) [36, 37]. According to the literature, pH values at 48 h are similar or slightly lower when compared to the pH at 24 h. The reason for this is that up to 24 h after slaughter, the glycogen in the muscle is rapidly converted into lactic acid resulting in pH decrease from 6.8–7.3 to 5.4–5.8. During the following 48 h, the meat goes through the process of maturation which is not accompanied by active glycogen conversion and therefore does not result in significant changes in pH values [38].

**Table 3:** Effect of *CTSB* g.72A>C SNP and *CTSL* g.143T>C SNP on meat quality traits. Data are presented as LSMeans ± SEM

Trait	Gene					
	*CTSB*			*CTSL*		
	g.72AA	g.72AC	*P*	g.143CC	g.143CT	*P*
Protein (g/100 g)	21.64 ± 0.19	21.76 ± 0.68	0.865	21.68 ± 1.51	20.50 ± 2.15	*0.023*
Intramuscular fat (g/100 g)	1.72 ± 0.98	1.24 ± 0.10	0.243	1.78 ± 1.02	1.68 ± 0.84	0.744
Shear force (g/cm^2^)	46.92 ± 1.16	39.85 ± 2.74	0.101	47.33 ± 1.26	42.48 ± 2.66	0.112
pH	5.47 ± 0.03	5.60 ± 0.02	0.099	5.46 ± 0.19	5.59 ± 0.16	*0.028*
Total water (g/100 g)	73.86 ± 3.08	74.28 ± 1.49	0.575	73.86 ± 1.86	74.74 ± 2.16	0.142
Ashes (g/100 g)	1.12 ± 0.01	1.16 ± 0.01	0.312	1.12 ± 0.09	1.06 ± 0.16	0.061

*P* values in italics show statistically significant differences

*CTSB* polymorphism had been previously studied with regards to growth and fattening traits in Italian Large White pigs [7, 26] showing a significant effect on backfat thickness [27]. These studies did not find effect of *CTSB* polymorphism on early muscle pH [7, 27]. More encouraging results were obtained in our study on *CTSL* (g.143C>T genotype, Table 3). On this locus, the total protein content was higher in pigs with g.143CC genotype when compared to CT animals (*P* = 0.023). In contrast, pigs with g.143CT genotype had a higher meat pH value then that for g.143CC pigs (*P* = 0.028). This tendency was also reported by [9] in Italian Large White pigs. However, it should be noted that in the present study, pH was measured at 48 h *post*-*mortem* whilst in the study by [9], pH was analysed at 2 and 24 h *post*-*mortem* which does not allow to make direct comparison.

The *GHRH* gene was selected for this study because of its relationship with growth and fat deposition, the two traits which have important implications for meat quality. Previous studies proved that injection of *GHRH* to pregnant sows increases piglets’ weight, both at birth and at weaning, and reduced the time required to reach the market [39, 40].

The present study established associations between *GHRH* polymorphisms, intramuscular fat content and meat tenderness, assessed as muscle shear force (Table 4). Pigs with *GHRH* BB genotype had a higher intramuscular fat content when compared to the animals with AB and AA genotypes. The relationship between this *GHRH* polymorphism and the carcass fat is well established. This can be illustrated by studies on Landrace pigs where the animals with *GHRH* BB genotype had thicker back fat when compared to AA and AB pigs [14]. Similarly, in an experimental group consisting of Yorkshire, Duroc and Landrace pigs, the animals with AA genotype had a 1.23 % higher meat percentage when compared to the animals with BB genotype [41].

**Table 4:** Effect of *GHRH Alu*I polymorphic site on meat quality traits

Trait	*GHRH*			
	AA	AB	BB	*P*
Protein (g/100 g)	22.69 ± 0.81^A^	21.65 ± 0.34^A^	21.17 ± 0.30^B^	0.18
Intramuscular fat (g/100 g)	1.23 ± 0.38^a,b^	1.41 ± 0.16^b^	1.90 ± 0.14^a^	*0.04*
Shear force (g/cm^2^)	44.16 ± 1.18^a,b^	40.86 ± 1.13^b^	49.50 ± 1.62^a^	*0.0004*
pH	5.52 ± 0.08	5.49 ± 0.03	5.50 ± 0.03	0.93
Total water (g/100 g)	73.53 ± 0.89	74.31 ± 0.37	74.04 ± 0.32	0.68
Ashes (g/100 g)	1.12 ± 0.05	1.12 ± 0.02	1.09 ± 0.02	0.39

Data shown as LSMeans ± SEM. Within line, mean with different superscript differ significantly at *P* < 0.05 (low case) and *P* < 0.1 (upper case)

*P* values in italics show statistically significant differences

Information available on pure-bred Large White pigs is fragmental and often inconsistent. Thus, although backfat thickness in pure-bred Large White has been reported to be affected by the *GHRH* genotype [42], the other authors [43] did not find effect of the *GHRH/Alu*I polymorphism on intramuscular fat and lean meat percentage, but consistently showed effect on average back fat thickness in this breed. This discrepancy might be related to the fact that [42] and [43] conducted their studies on different types of fat (subcutaneous and intramuscular fat respectively). Although positive correlation between intramuscular and subcutaneous fat content in pigs has been reported in a number of publications [44], there is an increasing number of studies suggesting that these two fat depots are regulated by different mechanisms and/or different genes [45, 46] and that the mechanisms of fat deposition might be breed-specific.

In the present study, the *GHRHAlu*I polymorphism has also significantly affected meat tenderness. Unexpectedly, our Large White population of *Alu*I-BB pigs, which had a higher intramuscular fat content, exhibited tougher meat (Table 4). The relationship between intramuscular fat content and meat tenderness is very much in dispute [47–49]. The latest reports indicate that in pigs tenderness positively correlates with intramuscular fat content values above 2 % [2], which is above the range of intramuscular fat in most of the animals used in present study. Moreover, intramuscular fat correlates with collagen content [49], which influences the mechanical strength of meat [50] and might explain the contradictory results between tenderness and intramuscular fat content across studies. The *GHRH* mutation had a significant dominant component for shear force and a high additive component for intramuscular fat content (Table 5). All these results are consistent with the fact that, in Ukrainian Large White pigs, allele A has a significant effect on favourable traits such as lower fat and more tender meat.

**Table 5:** Additive (A) and Dominant (D) components, allelic effects and allelic substitution effects of the *GHRH* and *LEPR* polymorphisms on the traits at the significance level of 0.05

Locus	Trait	Additive dominant model				
		*A*	*D*	*α*_*1*_	*α*_*2*_	*s* (1 → 2)
*GHRH*	Intramuscular fat (g/100 g)	−0.3350	−0.1550	−0.0718	0.1926	0.1322
	Shear force (g/cm^2^)	−0.4728	−8.2862*	−3.8972	−1.5115	−2.7043
*LEPR*	Ashes, g/100 g	−0.0650**	0.0650*	0.0130	−0.0293	−0.0211
	Intramuscular fat (g/100 g)	0.3110*	−0.9140**	0.0132	0.0043	−0.0045
	Shear force (g/cm^2^)	−2.8379	−6.1624**	0.8821	−1.5452	1.2137

* P < 0.05; ** P < 0 0.01; α_1_—effect allele 1; α_2_—effect allele2; s (1 → 2)—allelic substitution effect; For *GHRH*: *A* allele 1 and *B* allele 2; For *LEPR*: *C* allele 1 and *T* allele 2

The g.1987C>T polymorphism in the *LEPR* gene also had significant effects on intramuscular fat content and tenderness values in Ukrainian Large White pigs in this study. Animals with TT genotype, had a higher intramuscular fat content (*P* = 0.02) and, consequently, a lower amount of ashes (*P* = 0.02) and a tendency to a lower water content (*P* = 0.22) (Table 6). This is consistent with the data of literature that intramuscular fat content has significant negative relation with moisture and water content [51].

**Table 6:** Effect of *LEPR* SNP c.1987C>T mutation on meat quality traits

Trait	*LEPR*			
	g.1987CC	g.1987CT	g.1987TT	*P*
Protein (g/100 g)	21.75 ± 0.29	21.12 ± 0.34	20.97 ± 0.74	0.30
Intramuscular fat (g/100 g)	1.73 ± 0.16^b^	1.38 ± 0.19^b^	2.61 ± 0.40^a^	*0.02*
Shear force (g/cm^2^)	50.71 ± 1.69^a^	42.89 ± 1.72^b^	51.65 ± 5.04^a^	*0.02*
pH	5.45 ± 0.03	5.49 ± 0.03	5.53 ± 0.07	0.48
Total water (g/100 g)	74.01 ± 0.30^A^	74.62 ± 0.40^B^	73.10 ± 0.85^A,B^	0.22
Ashes (g/100 g)	1.12 ± 0.02^b^	1.12 ± 0.02^b^	0.99 ± 0.04^a^	*0.02*

Data shown as LSMeans ± SEM. Within line, mean with different superscript differ significantly at *P* < 0.05 (low case) and *P* < 0.1 (upper case)

*P* values in italics show statistically significant differences

Allele C behaves for this two traits in a complete dominant manner (Table 6), showing no difference between CC and CT genotypes. Accordingly, significant dominant effects were found for ash and fat content along with an additive component that highlights the differences reported in TT pigs (Table 5). The *LEPR* c.1987C>*T* mutation involves a L663F amino acid change in the coded protein that could be responsible for a reduction of the receptor function or signalling ability [52]. These data are consistent with results of other authors [17, 53] on Duroc x Iberian crosses and [18] on Iberian x Landraces cross-breed which demonstrated a higher association between the fat content and *LEPR* c.1987T allele when compared to c.1987C allele. On the other hand, there was a strong underdominant effect of this mutation on mechanical shear force, with CT pigs displaying the lower values (*P* = 0.02) and, therefore, a more tender meat (Table 6). This mutation has a significant dominant component for muscle shear force but the additive effect was not significant, emphasising the benefit of heterozygous CT animals, which would have, on the whole, more tender meat and lower fat content (Table 5).

To summarise, this study reports new data on allele variance in *CTSL*, *CTSB*, *CTSS* and *CTSK* genes as well as in *GHRH* and *LEPR* in pure-bred Ukrainian Large White pigs. In particular, a medium level of heterozygosity values was established for *GHRH* and *LEPR* genes which corresponded to very high levels of informativeness indexes. In contrast, cathepsins *CTSL*, *CTSB* and *CTSK* had a low level of heterozygosity, and *CTSS* did not segregate in this breed. Association studies demonstrated that intramuscular fat content and tenderness were affected by the allele variance in *GHRH* and *LEPR* but not by *CTSB* and *CTSL* genes. The *GHRH* results could be particularly relevant for the production of lean prime cuts as the A allele is associated with both, a lower meat fat content and, most importantly, better tenderness values, which are the two attributes highly regarded by consumers. The effect of this allele is in contrast with the overdominant manner by which the *LEPR* enhances tenderness and lowers fat content. Results of this study suggest that selective breeding towards *GHRH*/AA genotype would be particularly useful for improving meat quality characteristics in the production systems involving lean Large White lines with typical intramuscular fat content below 2 %. However, it should be noted that a relatively small number of animals is a limitation of this study and therefore further research and validation of these results on a large population is needed.
